# Beta-trace protein as a potential biomarker of residual renal function in patients undergoing peritoneal dialysis

**DOI:** 10.1186/s12882-021-02287-0

**Published:** 2021-03-11

**Authors:** Sebastian Schwab, Carola Ellen Kleine, Dominik Bös, Sylvie Bohmann, Christian P. Strassburg, Philipp Lutz, Rainer Peter Woitas

**Affiliations:** 1 Department of Internal Medicine I, University of Bonn, Bonn, Germany; 2 Institute of Experimental Immunology, Rheinische-Friedrichs-Wilhelms University of Bonn, Bonn, Germany; 3 KfH, Renal Center, Bonn, Germany; 4 Diaverum Deutschland GmbH, Munich, Germany

**Keywords:** Beta-trace protein, Low-molecular weight proteins, Peritoneal dialysis, Residual diuresis

## Abstract

**Background:**

Residual renal function is closely linked to quality of life, morbidity and mortality in dialysis patients. Beta-trace protein (BTP), a low molecular weight protein, has been suggested as marker of residual renal function, in particular in patients on hemodialysis. We hypothesized that BTP also serves as a marker of residual renal function in pertioneal dialysis patients.

**Methods:**

In this study 34 adult patients on peritoneal dialysis were included. BTP, creatinine, cystatin C and urea concentrations were analyzed simultaneously in serum and dialysate to calculate renal and peritoneal removal of the analytes.

**Results:**

In peritoneal dialysis patients with residual diuresis, mean serum BTP was 8.16 mg/l (SD ± 4.75 mg/l). BTP correlated inversely with residual diuresis (r_s_ = − 0.58, *p* < 0.001), residual creatinine clearance (Cl_Cr_) (r_s_ = − 0.69, *p* < 0.001) and total urea clearance (Cl_urea_) (r_s_ = − 0.56, *p* < 0.001). Mean peritoneal removal of BTP was 3.36 L/week/1.73m^2^ (SD ± 1.38) and mean renal removal 15.14 L/week/1.73m^2^ (SD ± 12.65) demonstrating a significant renal contribution to the total removal. Finally, serum BTP inversely correlated with alterations in residual diuresis (*r* = − 0.41, *p* = 0.035) and renal creatinine clearance over time (*r* = − 0.79, *p* = *p* < 0.001).

**Conclusion:**

BTP measurement in the serum may be a simple tool to assess residual renal function in peritoneal dialysis patients.

## Introduction

In patients with chronic kidney failure requiring dialysis, quality of life and survival are linked to residual renal function (RRF) [1–5]. To assess RRF, timed urea clearances or urea/creatinine clearances are considered the gold standard, but they rely on sampling both urine and serum. Commonly used serum markers, such as creatinine, cystatin C and urea do not reflect RRF accurately, because they are subject to clearance by the dialysis procedure. Moreover, all these low weight molecular proteins (LWMP) may be influenced by external factors. For instance, creatinine production depends on individual muscle mass, and is related to race, sex and age [6], while cystatin C concentration may be altered by smoking [7], obesity [8], diabetes [9], certain types of cancer [6], inflammation [10] or treatment with prednisone [11]. In addition, there is substantial evidence for a non-renal clearance of cystatin C [12].

Despite significant efforts, no reliable serum biomarker mirroring renal function has been identified yet. However Beta-trace protein (BTP) may be a promising candidate. BTP, with a molecular weight of 23- to 29-kDa, is a small molecule expressed in nearly all tissues involved in prostaglandin metabolism, such as the brain, retina, melanocytes, male reproductive organs, heart and kidney [13–16]. BTP is filtered freely in the kidneys with little if any tubular reabsorption and excreted in the urine. BTP serum levels correlate with GFR [17–19] and with residual kidney function (RRF) in end-stage renal disease (ESRD) [20, 21]. Due to its molecular weight, BTP is generally not removed by hemodialysis, but its elimination increases considerably in hemodiafiltration. In addition, elimination is largely depending on the applied dialysis membrane [22–25].

Little data is available concerning BTP in patients undergoing peritoneal dialysis (PD) [26, 27]. Recently, it was suggested that BTP, unlike other markers for glomerular filtration, is not cleared peritoneally at all [27].

The study presented here aims to characterize BTP removal in PD patients as well as to describe the relationship between serum BTP concentration, residual diuresis and residual creatinine clearance over time. We propose BTP as a useful tool to assess residual function in patients on maintenance peritoneal dialysis.

## Patients and methods

### Patients and treatment description

This prospective study included a total of 34 consecutive adult dialysis patients undergoing maintenance peritoneal dialysis. Of those, 29 had residual diuresis > 200 ml/24 h.

Dialysis efficiency was estimated using Kt/V ratio. All maintenance dialysis patients were on regular dialysis treatment. PD patients were on automated PD (APD, *n* = 2) or continuous PD (CAPD, *n* = 32). Evaluation of peritoneal membrane characteristics was quarterly performed by using the modified peritoneal equilibrium test (PET). Patients were then categorized into a fast and slow transport type. Patients with a diuresis less than 200 ml were assigned to the anuria group.

### Parameters and assays

All patients were routinely subjected to blood sample collections to quantify PD treatment adequacy and to a peritoneal adequacy test.

BTP, creatinine, cystatin C and urea concentrations were analyzed simultaneously in serum, dialysate and urine. Levels of BTP and cystatin C were analyzed by immunonephelometry, levels of urea by photometry, and creatinine concentration by a modification of the Jaffé method. Creatinine Clearance (Cl_Cr_) was adjusted to a standard body surface area of 1,73 m^2^.

Serum creatinine was measured using a modified Jaffé method; urea by a standard clinical analyzer. Serum cystatin C and BTP were analyzed by fully automated, latex-enhanced immunonephelometry (N latex Cystatin C, N latex BTP, respectively, on a Nephelometer II; Siemens, Erlangen, Germany).

Data on the residual diuresis were documented quarterly. Volumes of residual diuresis were determined by means of a 24-h urine collection as routinely performed at home quarterly.

### Renal and peritoneal elimination of LWMP

The calculation of residual renal creatinine clearance was performed and normalized to body surface area (BSA) as follows:

$$ \mathrm{Renal}\ {\mathrm{Clearance}}_{\mathrm{creatinine}}=\left({\mathrm{volume}}_{\mathrm{urine}}\times {\mathrm{concentration}}_{\left(\mathrm{creatinine}\ \mathrm{urine}\right)}\right) / \left({\mathrm{concentration}}_{\left(\mathrm{creatinine}\ \mathrm{serum}\right)}\times \mathrm{collection}\ {\mathrm{time}}_{\left(\mathrm{minutes}\right)}\right)\ast 1,73 / \mathrm{BSA} $$

In PD patients, weekly renal and peritoneal elimination of low molecular weight proteins were calculated and normalized to BSA as follows:

Weekly peritoneal clearance of LWMP:

$$ 7\ \mathrm{x}\ \left({\mathrm{concentration}}_{\mathrm{Dialysate}}\times {\mathrm{volume}}_{\mathrm{Dialysate}} / {\mathrm{concentration}}_{\mathrm{Serum}}\right)\ast 1,73/\mathrm{BSA}\left(\mathrm{inL}/\mathrm{week}/{\mathrm{m}}^2\right) $$

Weekly renal clearance:

$$ 7\ \mathrm{x}\ \left({\mathrm{concentration}}_{\mathrm{Urine}}\times {\mathrm{volume}}_{\mathrm{Diuresis}} / {\mathrm{concentration}}_{\mathrm{Serum}}\right)\ast 1,73/\mathrm{BSA}\left(\mathrm{inL}/\mathrm{week}/{\mathrm{m}}^2\right) $$

Total clearance was calculated as the sum of the peritoneal and the renal clearance.

### Calculation of correlation between BTP and diuresis over time

To analyze a correlation between loss of diuresis and increase in serum LWMP over time all patients with residual diuresis and a minimum follow-up of 12 months were included.

Differences between the last and the first BTP serum value as well as the last and first diuresis volume were calculated and divided by the amount of follow-up quarters.

$$ \left({{\mathrm{last}}_{\mathrm{BTP}}}_{\mathrm{serum}}-{{\mathrm{first}}_{\mathrm{BTP}}}_{\mathrm{serum}}\ \right) / \mathrm{amount}\ \mathrm{of}\ \mathrm{quarters} $$

$$ \left({\mathrm{last}}_{\mathrm{diuresis}\ \mathrm{in}\ \mathrm{ml}}-{\mathrm{first}}_{\mathrm{diuresis}\ \mathrm{in}\ \mathrm{ml}}\right) / \mathrm{amount}\ \mathrm{of}\ \mathrm{quarters} $$

In addition, we also investigated likewise the correlation between decline in creatinine clearance and increase in serum BTP over time.

$$ \left({{\mathrm{last}}_{\mathrm{BTP}}}_{\mathrm{serum}}-{{\mathrm{first}}_{\mathrm{BTP}}}_{\mathrm{serum}}\right) / \mathrm{amount}\ \mathrm{of}\ \mathrm{quarters} $$

$$ \left({\mathrm{last}}_{\mathrm{CrCl}}-{\mathrm{first}}_{\mathrm{CrCl}}\right) / \mathrm{amount}\ \mathrm{of}\ \mathrm{quarters} $$

Afterwards, correlation between the values was calculated.

### Statistics

Statistical analysis was performed with GraphPad Prism 6 (La Jolla, USA). Results are given as mean with standard deviation (SD) for quantitative variables. T-tests or one-way ANOVA were used for the comparison of quantitative values.

Spearman rank coefficient was used to test the correlation between two variables. By plotting a receiver operating characteristic (ROC) curve, we determined the best cut-off value for a residual renal creatinine clearance of 7 ml / min / 1.73m^2^. Two-sided p-levels < 0.05 were considered statistically significant.

### Ethics approval

The study was approved by the local ethics committee, the ethics committee of the medical faculty of the university of Bonn – number 159/17, and informed consent was obtained from all patients enrolled into the study. The study was performed in accordance with the relevant guidelines and regulations.

## Results

### General characteristics of patients and treatment modality

In total, 34 patients (15 female and 19 male) were included into the study. Patients clinical and biochemical characteristics are listed in Table 1. Major causes of ESRD were glomerulonephritis, renovascular disease and interstitial nephritis. Of the patients with residual diuresis (*n* = 29), mean serum BTP was 8.16 mg/l (SD ± 4.75 mg/l). Serum BTP level correlated with serum creatinine (r_s_ = 0.8, *p* < 0.001), serum cystatin C (r_s_ = 0.79, *p* < 0.001) and serum urea concentration (r_s_ = 0.12, *p* = 0.15). There was no significant correlation between serum levels of BTP and sex (*r* = 0.11; *p* = 0.6), body mass index (*r* = − 0.24; *p* = 0.34) or age (r_s_ = − 0.3, *p* = 0.17).

**Table 1:** General characteristics of patients

patients in total	29
**sex (male / female)**	**18/11**
**age (years)**	**52.7** ± **12.15**
**body weight (kg)**	**81.16 (SD** ± **14.96)**
**body mass index**	**26.88 (SD** ± **4.55)**
**residual diuresis (ml)**	**1374 (SD** ± **913)**
**PD regime (CAPD)**	**29**
**serum BTP (mg/L)**	**8.16 (SD** ± **4.75)**
**serum Cystatin C (mg/L)**	**5.78 (SD** ± **1.6)**
**serum urea (mg/dL)**	**123.5 (SD** ± **70.3)**
**serum creatinine (mg/dL)**	**8.83 (SD** ± **3.59)**
**dialysate volume (L)**	**8.86 (SD** ± **1.9)**
**dialysate BTP (mg/L)**	**0.54 (SD** ± **0.34)**
**dialysate Cystatin C (mg/L)**	**0.64 (SD** ± **0.35)**
**dialysate urea (mg/dL)**	**107.9 (SD** ± **42.01)**
**dialysate creatinine (mg/dL)**	**7.03 (SD** ± **9.4)**
**urine BTP (mg/L)**	**14.7 (SD** ± **9.58)**
**urine Cystatin C (mg/L)**	**5.44 (SD** ± **4.53)**
**urine urea (mg/dL)**	**382.89 (SD** ± **232.2)**
**urine creatinine (mg/dL)**	**51.12 (SD** ± **36.81)**
**weekly Kt/V**	**2.28 (SD** ± **0.76)**
**weekly peritoneal Kt/V**	**1.4 (SD** ± **0.49)**
**weekly renal Kt/V**	**0.86 (SD** ± **0.65)**
**total creatinine clearance (L/week/1,73 m2)**	**91 (SD** ± **51.61)**
**renal creatinine clearance (L/week/1,73 m2)**	**54.82 (SD** ± **52.87)**
**peritoneal creatinine clearance (L/week/1,73 m2)**	**34.29 (SD** ± **12.49)**

General characteristics of patients and treatment modality. Data are given as mean and standard deviation

### LWMP values in serum and dialysate in patients with and without residual diuresis

Serum BTP concentration was higher in anuric patients (Residual diuresis: mean 8.16 mg/L, SD ± 4.75 mg/L; anuria: mean 12.2 mg/L, SD ± 3.84 mg/L, *p* < 0.05). Anuric PD patients had a mean dialysate BTP concentration of 0.6 mg/l (SD ± 0.32), which was comparable to PD patients with residual diuresis (mean 0.54 mg/l (SD ± 0.34), but dialysis volume per day was considerably higher in the anuric group (Residual diuresis: mean 8.86 L/day, SD ±1.9 versus anuria: mean 13.97 L/day, SD ± 6.17 L/day). In contrast to creatinine, cystatin C and urea, only serum BTP was significantly higher in anuric patients compared to patients with residual diuresis. Anuric patients were excluded from the following analyses.

### Renal and peritoneal clearance of LWMP

**Fig. 1:**
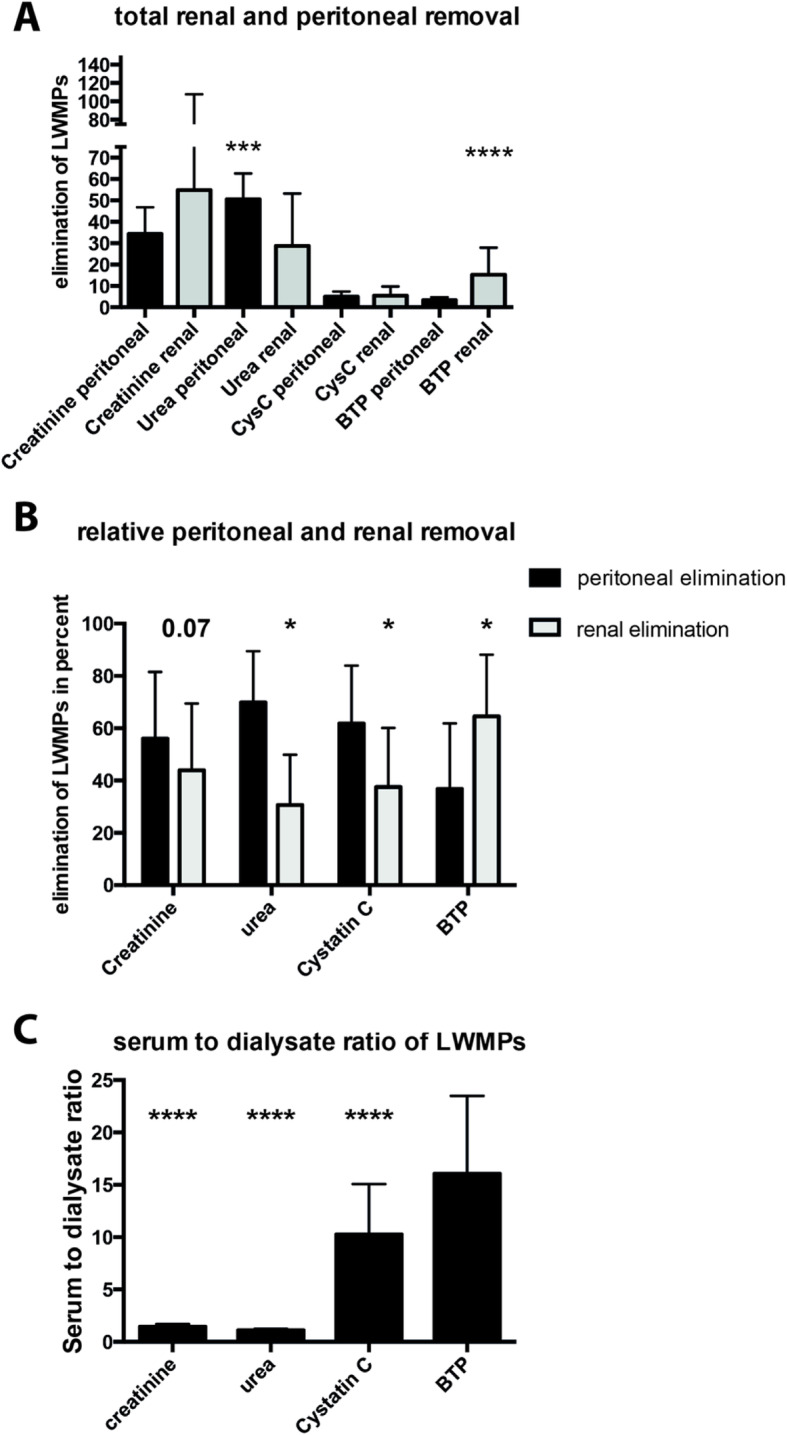
Renal and peritoneal elimination of analytes in patients with residual diuresis. **a** – total renal and peritoneal removal of analytes [(creatinine and urea in L / week / m^2^); (cystatin C (CysC) and BTP in L / week / m^2^)]; **b** – percentage expression of renal and peritoneal elimination. **c** – Quotient of serum and dialysate values for respective analyte. Data are given as mean and standard deviation. *P*-values < 0.05 were considered statistically significant. *p* < 0.05 = *; *p* < 0.001 = **; *p* < 0.0001 = ***; *p* < 0.00001 = ****

Mean peritoneal removal of BTP was 3.36 L/week/1.73m^2^ (SD ± 1.38) and mean renal removal 15.14 L/week/1.73m^2^ (SD ± 12.65; *p* < 0.01) demonstrating a minor peritoneal removal (Fig. 1, Table 2). Renal and peritoneal removal of cystatin C and creatinine showed no significant differences. BTP was the only solute demonstrating a higher renal than peritoneal elimination, which was reflected by a significantly higher ratio of BTP _Serum_ to BTP _Dialysate_ (Fig. 1 a-c).

**Table 2:** Renal and peritoneal removal of LWMPs. Values are given as mean and standard deviation. Only data of patients with residual diuresis were included

	Creatinine (L/week/1,73m^**2**^)	Urea (L/week/1,73m^**2**^)	Cystatin C (L/week/1,73m^**2**^)	BTP (L/week/1,73m^**2**^)
**patients with diuresis**				
**total clearance / week**	**91 (**± **51.61)**	**76.83 (**± **20.85.9)**	**10.63 (**± **5.68)**	**18.35 (**± **13.37)**
**renal removal / week**	**54.82 (**± **52.87)**	**28.68 (**± **24.51)**	**5.43 (**± **4.29)**	**15.14 (**± **12.65)**
**peritoneal removal / week**	**34.29 (**± **12.49)**	**50.53 (**± **12.1)**	**4.95 (**± **2.48)**	**3.36 (**± **1.38)**

### LWMPs and residual renal function

Mean residual creatinine-clearance was 6.43 ml/min/1.73m^2^ (SD ± 0.41), mean residual urea clearance (KRU) 2.97 ml/min/1.73m^2^ (SD ± 2.42), mean residual creatinine-urea clearance (KRU/crea) 4.11 ml/min/1.73m^2^ (SD ± 3.65) and mean BTP clearance 1.89 ml/min/1.73 m^2^ (SD ± 1.56) in patients with residual diuresis.

**Fig. 2:**
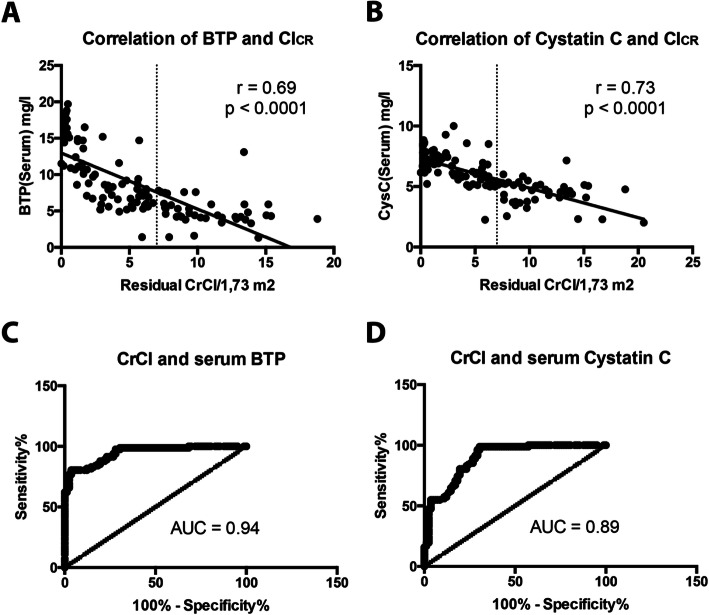
Correlation of serum analytes and their predictive value. Figure 2: Correlation of serum analytes and residual renal creatinine clearance (ClCR). **a** – Serum BTP and ClCR; **b** - Serum Cystatin C and ClCR. Receiver operating characteristic (ROC) curve for prediction of a creatinine clearance < 7 ml / min based on serum level of BTP and Cystatin C. **c** – ROC analysis for BTP; **d** – ROC analysis for Cystatin C. AUC (Area under the curve): BTP: 0.94; Cystatin C: 0.89

Serum BTP correlated inversely with residual diuresis (r_s_ = − 0.58, *p* < 0.001), residual creatinine clearance (Cl_Cr_) (r_s_ = − 0.69, *p* < 0.0001) and urea clearance (Cl_urea_) (r_s_ = − 0.56, *p* < 0.001). The correlation of the other investigated analytes with residual diuresis was lower (Creatinine: *r* = − 0.5, *p* < 0.001; Cystatin C: *r* = − 0.54, *p* = *p* < 0.001; Urea: *r* = − 0.08, *p* = 0.36). Cystatin C correlated equally well with Cl_Cr_ in comparison to BTP [r_s_ = − 0.73, *p* < 0.0001) (Fig. 2)].

By applying ROC analysis, a serum BTP concentration of 6.7 mg / l was identified as cut-off value to distinguish between a residual renal creatinine clearance of < 7 ml / min / 1.73 m^2^ and ≥ 7 ml / min / 1.73 m^2^ with a sensitivity of 76.8% (95% CI: 66.2–85.4) and a specificity of 97.6% (91.5–99.7)]. The positive and negative predictive value were calculated as 90.2 and 87.9%, respectively.

In comparison, BTP performed better than Cystatin C with an AUROC of 0.94 compared to 0.89. A serum cystatin C concentration of 5 mg / l was detected to differentiate between the clearance treshold of 7 ml / min / 1.73m^2^ with a sensitivity of 96.3% (95% CI: 89.7–99.2), a specificity of 70.3% (95% CI: 58.4–79.2), a positive predictive value of 81.4% and a negative predictive value of 46.2% (Fig. 2).

**Fig. 3:**
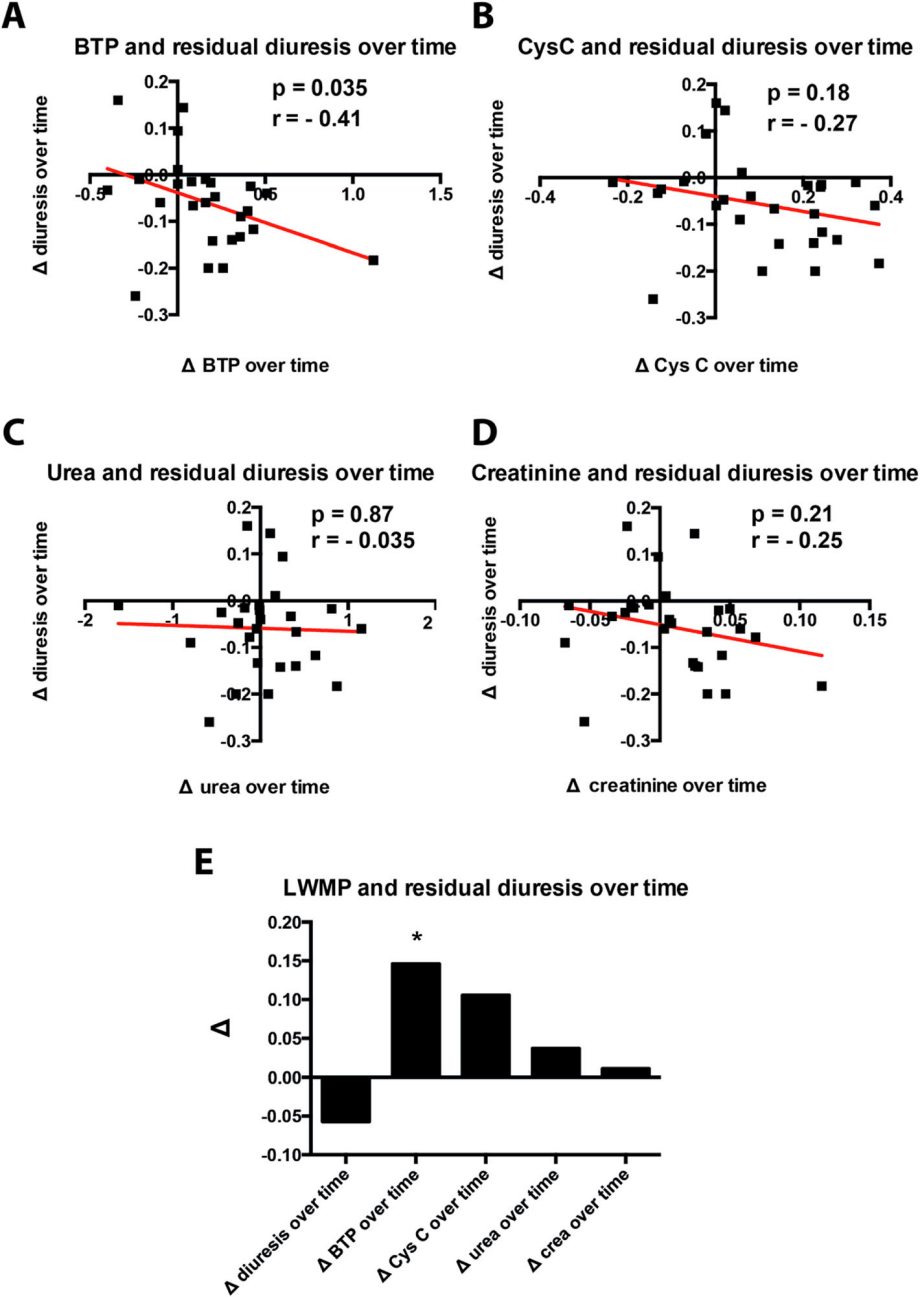
Serum analytes and their correlation with residual diuresis over time. All patients with residual diuresis who were followed up for at least 12 months were included. Correlation between the last and the first BTP serum value as well as the last and first diuresis volume were divided by the amount of follow-up quarters. **a**- BTP; **b**- Cystatin C (CysC); **c**- urea; **d** – creatinine; **e** – overview over all investigated parameters. Data are given as mean and standard deviation. *P*-values < 0.05 were considered statistically significant; *p* < 0.05 = *

To assess changes of residual diuresis over time and corresponding serum levels of analytes, we calculated the difference between the last and first diuresis volume as well as BTP serum values and divided it by the amount of follow-up quarters. In contrast to the other markers, change in serum BTP correlated inversely with change in residual diuresis (*r* = − 0.41, *p* = 0.035) over time (Fig. 3).

Likewise, we further assessed the correlation between changes in renal creatinine clearance and serum levels of LWMPs.

**Fig. 4:**
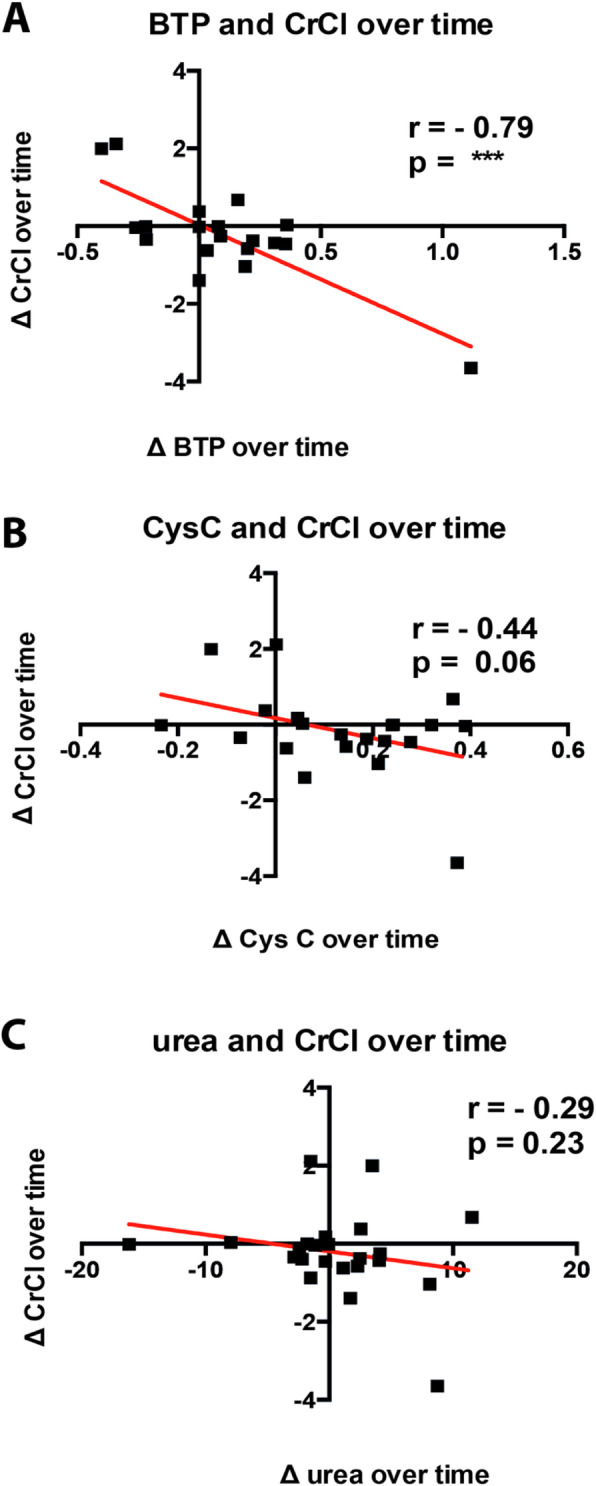
Serum analytes and their correlation with creatinine clearance over time. All patients with residual diuresis who were followed up for at least 12 months were included. Correlation between the last and the first BTP serum value as well as the last and first creatinine clearance were divided by the amount of follow-up quarters. **a**- BTP; **b**- Cystatin C (CysC); **c**- urea. Data are given as mean and standard deviation. *P*-values < 0.05 were considered statistically significant; *p* < 0.05 = *; *p* < 0.001 = **; *p* < 0.0001 = ***; *p* < 0.00001 = ****

The increase in serum BTP and the decline of renal creatinine clearance over time correlated well ((*r* = − 0.79, *p* = < 0.001). In contrast, changes of serum cystatin C as well as of serum urea did not correlate with residual creatinine clearance over time (Fig. 4).

### Peritoneal clearance of LWMP in fast and slow transporters

**Fig. 5:**
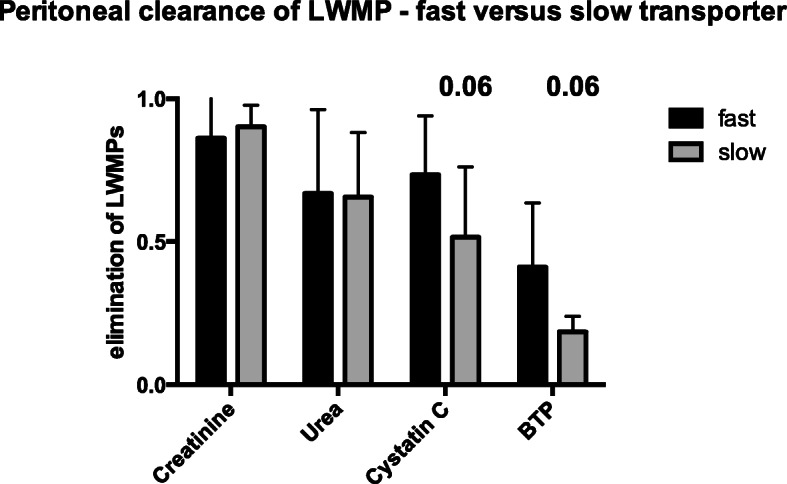
Peritoneal clearance in fast and slow peritoneal transporter. The cohort was divided into fast (*n* = 16) and slow peritoneal transporter (*n* = 5). Mean and standard deviation of peritoneal elimination are given

Fast transporters (*n* = 16) exhibited a higher relative peritoneal clearance for BTP compared to slow transporters (*n* = 5) (fast: 41% SD ± 22% versus slow 19.9% SD ± 5.4%, *p* = 0.06), We did not note similar differences for creatinine and urea, whereas relative peritoneal elimination of cystatin C showed similar results as for BTP elimination (*p* = 0.06; Fig. 5).

## Discussion

RRF is associated with better survival in dialysis patients [4]. To date, RRF is generally expressed as urinary clearance of urea (United States) or the average of urea and creatinine clearance (European best practice guidelines) calculated from interdialytic urine collection and then corrected to 1.73m^2^ [28]. These methods need a timed urine collection, which is regarded a burden for patients and staff and therefore prone to failure or unsound results. Thus, there is a clinical need for simpler methods assessing RRF, such as by endogenous serum markers, in particular BTP and cystatin C [29–32]. Yet, studies of serum markers in PD are lacking. To the best of our knowledge, there are only a few studies investigating BTP as potential biomarker of RRF in PD patients [26, 27, 33].

In our study cohort, serum BTP levels correlated well with established biomarkers of renal function such as serum creatinine, serum cystatin C and total Cl_Cr_.

However, in contrast to the other parameters, only BTP showed a considerably high renal elimination during peritoneal dialysis. In fact, the renal elimination of BTP exceeds the peritoneal elimination, whereas all other investigated surrogate parameters are predominantly eliminated peritoneally (Fig. 1, Table 2). In line, serum BTP concentration was significantly higher in the anuric group than in patients with RRF, although the considerable difference in dialysis volume between the groups might be a confounding factor.

Serum BTP concentration has been reported to reflect RRF in patients undergoing hemodialysis [22, 23]. In a previous study, we demonstrated that, except for postdilution HDF, the applied dialysis procedures do not affect BTP levels significantly. However, with increasing permeability of the applied membrane, the reliability of BTP as a marker of RRF tends to decrease [23, 25]. For PD patients, peritoneal clearance of small molecules, such as creatinine, was described to be largely dependent on the number of dialysate exchanges. In contrast, peritoneal clearance of middle molecules, such as BTP, is mainly depending on the total dwell hours of PD [34]. Contrary to our findings a previous study reported no BTP removal by PD [27]. However, this study was comprised of only two patients on CAPD treatment, whereas in our study all patients with residual diuresis underwent CAPD. Summarizing our and previous studies’ findings on BTP in PD, peritoneal elimination of BTP is likely to depend on the PD modality and to increase with longer dwell time. Nevertheless we propose BTP as a good marker to describe RRF in PD patients due to its superiority over the established markers including creatinine, urea and cystatin C [27, 34].

In addition, our results indicate that peritoneal BTP clearance may depend on the transporter status, as peritoneal clearance was lower in the group of slow transporters. Nevertheless, BTP is a valuable marker of RRF independent of transporter statues because the majority of our cohort was classified as fast transporters.

We could not demonstrate any gender-associated difference in serum BTP concentrations, which is contrary to a study in kidney transplanted patients [20]. Probably, the effect of kidney function on BTP levels is much more pronounced than the effect of gender, so that gender differences do not become apparent in studies including patient with end-stage renal disease.

Although elimination ratio of LWMP depends on several factors, molecular mass is a factor of outstanding importance in the elimination over membranes. Molecular mass of BTP (23–29 kDa) is larger than cystatin C with a molecular weight of 13.3 kDa, which can partially explain why peritoneal clearance of cystatin C was higher compared to BTP. Cystatin C has shown a considerable non-renal clearance of 22.3 ml/min/1.73m^2^ that greatly exceeds its renal clearance in advanced renal failure [12, 35], which might also explain why BTP performed better as marker of RRF in our study.

In addition to the established association of BTP to residual diuresis in end-stage renal disease, we present longitudinal data on the association of residual diuresis during a follow-up period of at least 12 months. In contrast to the other serum markers, change in BTP serum concentration correlated inversely and significantly with the change of diuresis over time.

Similar results could be demonstrated for the correlation between the increase in serum BTP and the decline of residual creatinine clearance over time, strengthening the concept that BTP might serve as surrogate marker for RRF and suggesting that rising levels of serum BTP over time indicate worsening kidney function.

Our data should be interpreted with a note of caution since our investigation was limited to a rather small study population consisting mostly of Caucasians. However, two other studies indicated that serum BTP might be superior to other serum markers of RRF [33, 34]. Clearly, replication in a larger cohort of PD patients with longer follow up time is needed to define BTP cut-off values for clinical practice.

In addition, our cohort size precludes reliable assessment of clinical outcomes. In a larger cohort, a BTP-based eGFR should be determined and associated with all-cause mortality, cardiovascular mortality and hospitalization, which might reveal if the statistical superiority of BTP over cystatin C translates into a better clinical prognostic prediction.

In summary, our study suggests BTP as a reliable marker of RRF in patients PD. Based on the demonstrated elimination rates of the investigated analytes, BTP seems superior to other markers since it shows a relatively moderate peritoneal elimination. In addition, changes in serum BTP over time reflect changes in residual diuresis. Further investigations are warranted to elucidate the predictive value of testing serum levels of BTP to assess RRF.

## Data Availability

The datasets used and/or analysed during the current study are available from the corresponding author on reasonable request.

## Abbreviations

BSA: Body surface area
BTP: Beta-trace protein
ESRD: End stage renal disease
LWMP: Low weight molecular protein
kDa: Kilo dalton
GFR: Glomerular filtration rate
RRF: Residual renal function
KRU: Urea clearance
KRU/crea: Creatinine-urea clearance
SD: Standard deviation

## Reference intervals of analytes in our laboratory

BTP: 0.39–0.74 mg/l
cystatin: C 0.61–0.95 mg/l
creatinine: 0.7–1.3 mg/dl
urea: 16.6–48.5 mg/dl
